# Targeting mitochondrial Kv1.3 enables precise autoreactive T cell therapy for multiple sclerosis

**DOI:** 10.1038/s44321-025-00306-3

**Published:** 2025-09-29

**Authors:** Stefano Pluchino, Cory M Willis

**Affiliations:** Department of Clinical Neurosciences and NIHR Biomedical Research Centre, University of Cambridge, Cambridge, UK

**Keywords:** Immunology

## Abstract

Pluchino and Willis discuss a study by Szabo et al (in this issue of *EMBO Mol Med*) that reveals a novel therapy to treat multiple sclerosis by targeting the mitochondrial Kv1.3 channel in autoreactive T cells.

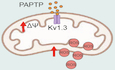

## Teaser

Multiple sclerosis (MS), a chronic autoimmune disorder, is characterized by the infiltration of autoreactive T cells into the central nervous system (CNS), leading to myelin destruction and neurological deficits. Current therapies often lack specificity, resulting in broad immunosuppression and potential side effects. In this issue of *EMBO Molecular Medicine*, Angi, Varanita and colleagues (Angi et al, 2025) identify a novel therapeutic strategy in targeting the mitochondrial Kv1.3 channel in autoreactive T cells, offering a more selective approach to mitigate MS-like pathology.

Multiple sclerosis (MS) is a debilitating autoimmune disorder affecting ~2.9 million individuals worldwide, targeting the central nervous system (CNS) and leading to significant neurological disability (Koch-Henriksen and Magyari, 2021). Relapsing-remitting MS (RR-MS), the most common early form of the disease, is characterized by periods of new or worsening symptoms (*relapses*) followed by periods of recovery (*remission*). The pathogenesis of RR-MS is largely driven by myelin-specific autoreactive CD4^+^ T cells, which are activated in the periphery by antigen-presenting cells (APCs) (Fransen et al, 2020). These activated T cells then cross the blood-brain barrier, enter the CNS, and initiate a cascade of inflammatory events, including activation of resident microglia and infiltrating macrophages. The resulting inflammatory response leads to the destruction of the myelin sheath surrounding neuronal axons, causing impaired nerve conduction and progressive motor deficits.

**Figure 1:**
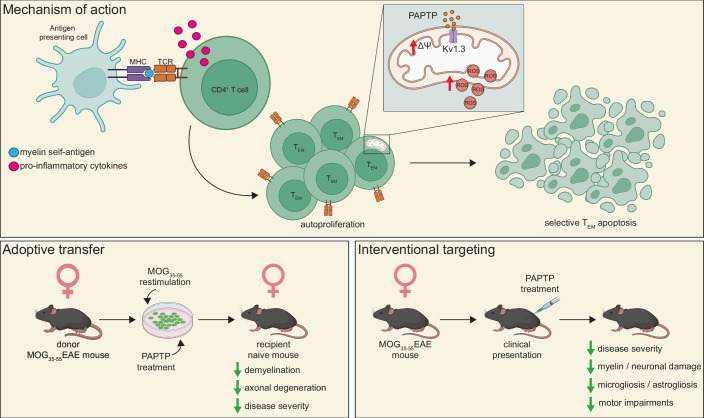
Autoreactive T-cell activation and the therapeutic action of PAPTP in MS-like disease. Myelin self-antigen presentation by antigen-presenting cells (APCs) to naive CD4^+^ T cells triggers their autoproliferation and activation into effector memory T (T_EM_) cells. Treatment with PAPTP blocks the mitochondrial Kv1.3 (mitoKv1.3) ion channel, leading to mitochondrial hyperpolarization (ΔΨ), increased reactive oxygen species (ROS) production, and subsequent apoptosis of T_EM_ cells. The adoptive transfer EAE model involves the re-stimulation and transfer of myelin-specific T cells from female donor mice to naive recipient mice. Pre-treatment of donor T cells with PAPTP prevents disease onset and reduces myelin and axonal damage in recipient mice. In the interventional EAE model, the systemic PAPTP administration (4 nmol/gram of body weight, intraperitoneally every 48 h for 20 days, initiated upon first presentation of clinical symptoms) alleviates clinical severity, motor deficits, and inflammation while preserving myelin and axonal integrity. Created with BioRender.com.

Chronically activated CD4^+^ T effector memory (T_EM_) cells are key players in MS pathogenesis, representing the major infiltrating cell type in MS brains (Rus et al, 2005). Notably, these cells exhibit high expression of the Kv1.3 potassium ion channel in the plasma membrane (PM). The Kv1.3 channel is crucial for T-cell proliferation, maintaining the negative membrane potential required for Ca^2+^ influx during cell activation (Fig. 1). While selective peptide toxin-based inhibitors targeting PM Kv1.3 exist, they primarily decrease T-cell proliferation without inducing cell death. Intriguingly, the Kv1.3 channel is also found in the inner mitochondrial membrane (mitoKv1.3), where its expression positively correlates with PM Kv1.3 levels in both human and mouse T cells (Capera et al, 2022; Szabó et al, 2008; Szabò et al, 2005).

Previously, this group developed a membrane-permeable derivative of the PM Kv1.3 inhibitor PAP-1, termed PAPTP, designed to selectively target mitoKv1.3 (Leanza et al, 2017). PAPTP’s effect is specific and dependent on the expression of mitoKv1.3 (Prosdocimi et al, 2024), leading to hyperpolarization of the inner mitochondrial membrane and subsequent increases in mitochondrial reactive oxygen species (ROS) production and cytochrome c release, ultimately inducing apoptosis (Leanza et al, 2017; Severin et al, 2022). Indeed, PAPTP has demonstrated selective ROS-mediated apoptosis of cancer cells in vivo (Leanza et al, 2017) and cytochrome c release and apoptosis in pathologic Kv1.3 high B cells in a murine model of chronic B cell lymphocytic leukemia (Severin et al, 2022) without harming other cell types.

While potent disease-modifying therapies (DMTs) exist for RR-MS, their lack of specificity and broad immunosuppression highlight the need for more targeted approaches. Addressing this challenge, Angi, Varanita et al, report in *EMBO Molecular Medicine* (Angi et al, 2025) on the ability of PAPTP to selectively target and kill autoreactive/autoproliferative CD4^+^ T_EM_ in the peripheral blood of people with MS and in experimental autoimmune encephalomyelitis (EAE), an animal model of MS-like disease.

To effectively induce apoptosis, PAPTP relies on target cells expressing high levels of Kv1.3 and elevated intracellular ROS. As Kv1.3 is highly expressed in T_EM_ cells in people with MS (Beeton et al, 2006), and mitochondrial metabolism is altered in these cells (De Biasi et al, 2019), the authors sought to determine whether ROS levels were also altered. Indeed, profiling of peripheral blood mononuclear cells from people with RR-MS positive for the HLA-DRB1*15:01 susceptibility gene associated with heightened T-cell autoproliferation (Jelcic et al, 2018; Mohme et al, 2013) and undergoing treatment with natalizumab revealed significantly increased ROS levels in CD4^+^/CD25^+^/CCR7^-^ T_EM_, compared to CD4^+^/CD25^+^/CCR7^+^ naive and central memory T cells (T_CM_).

Next, the authors determined whether PAPTP was sufficient to further increase ROS levels, which is critical for triggering apoptosis. PAPTP treatment (1 μM) augmented ROS levels in both T_EM_ and naive/T_CM_ cells, with the most pronounced effect observed in T_EM_ cells. However, the increased ROS levels observed in naive/T_CM_ cells treated with PAPTP did not induce apoptosis, suggesting that PAPTP-driven ROS levels must pass a critical threshold to induce apoptosis. The authors confirmed that the increased mitochondrial membrane potential and apoptosis in T_EM_ cells treated with PAPTP were specific to the inhibition of mitoKv1.3, not PM Kv1.3. Furthermore, PAPTP treatment did not significantly alter the relative proportions of CD3^+^ T cells, CD19^+^ B cells, CD4^+^ T helper cells, and CD8^+^ cytotoxic T cells. These findings suggest that PAPTP can selectively target and kill T_EM_ cells to improve MS pathology.

To further investigate the impact of PAPTP on MS pathology, the authors turned to mice. They discovered that deletion of Kv1.3 in mouse splenocytes prevented PAPTP-induced apoptosis, an effect that could be rescued by transfecting cells with a mitoKv1.3 construct. Next, they tested whether PAPTP treatment of myelin-specific T_EM_ cells could influence the clinical disease trajectory in the adoptive transfer model of EAE (Fig. 1). Mice receiving PAPTP-treated T cells displayed no clinical deficits and exhibited significantly reduced axonal degeneration, demyelination, and astrogliosis. Immunophenotyping analysis revealed that PAPTP treatment led to selective apoptosis in T_EM_ cells while preserving naive/T_CM_ cells.

To assess the potential of PAPTP to reverse existing MS-like pathology, the authors initiated PAPTP treatment in mice with established EAE after the first signs of clinical presentation. Mice received an intraperitoneal injection of PAPTP every 48 h for 20 days. Consistent with the adoptive transfer experiments, PAPTP treatment significantly reduced clinical deficits, axonal degeneration, demyelination, astrogliosis, and microgliosis. Immunophenotyping analysis confirmed selective apoptosis of autoreactive T_EM_ cells in PAPTP-treated mice without affecting other immune populations. Notably, PAPTP does not efficiently cross the blood-brain barrier, so its effects are largely limited to the periphery. Long-term treatment with PAPTP did not result in generalized immune suppression, liver or spleen toxicity, or depletion of red or white blood cells or platelets.

These findings present a compelling case for targeting T_EM_ cells as a therapeutic strategy—not only for people with RR-MS (Fig. 1), but also potentially in other neuroinflammatory disorders involving T cell-mediated demyelination.

While the evidence is promising, certain limitations warrant further study for clinical translation. First, more extensive immunophenotyping of the patient's blood is warranted. While the current study looked broadly at T and B cells and macrophages, other immune cells involved in MS pathology were overlooked (e.g., dendritic cells and neutrophils). Applying high-dimensional flow cyometry (e.g., CyTOF or spectral) would allow for a more comprehensive analysis of the specificity of PAPTP to T_EM_. Second, immunophenotyping was performed on a relatively small subset of RR-MS patients with the HLA-DRB1*15:01 susceptibility gene who were undergoing natalizumab treatment, raising questions about the generalizability of these findings. Analyzing blood samples from a more diverse cross-section of RR-MS patients would be more informative. Finally, given the large number of DMTs available, clinical trials would need to compare PAPTP treatment to current frontline DMTs to determine whether PAPTP offers improved benefits (e.g., patient-reported outcomes vs clinically measurable outcomes).

In conclusion, Angi et al (Angi et al, 2025) present a compelling study demonstrating that selectively targeting the mitoKv1.3 channel in chronically autoreactive TEM cells with PAPTP can reduce MS-associated inflammation. While further research into the selectivity of PAPTP for TEM cells and its efficacy in the broader RR-MS population is necessary, these findings offer a promising avenue for the development of a new targeted T-cell therapy to improve the quality of life and outcomes in people with MS.
